# Acute vs. Chronic vs. Cyclic Hypoxia: Their Differential Dynamics, Molecular Mechanisms, and Effects on Tumor Progression

**DOI:** 10.3390/biom9080339

**Published:** 2019-08-03

**Authors:** Kritika Saxena, Mohit Kumar Jolly

**Affiliations:** Centre for BioSystems Science and Engineering, Indian Institute of Science, Bangalore 560012, India

**Keywords:** cyclic hypoxia, intermittent hypoxia, obstructive sleep apnea, HIF-1α signaling, acute hypoxia, chronic hypoxia, mathematical modeling

## Abstract

Hypoxia has been shown to increase the aggressiveness and severity of tumor progression. Along with chronic and acute hypoxic regions, solid tumors contain regions of cycling hypoxia (also called intermittent hypoxia or IH). Cyclic hypoxia is mimicked *in vitro* and *in vivo* by periodic exposure to cycles of hypoxia and reoxygenation (H–R cycles). Compared to chronic hypoxia, cyclic hypoxia has been shown to augment various hallmarks of cancer to a greater extent: angiogenesis, immune evasion, metastasis, survival etc. Cycling hypoxia has also been shown to be the major contributing factor in increasing the risk of cancer in obstructive sleep apnea (OSA) patients. Here, we first compare and contrast the effects of acute, chronic and intermittent hypoxia in terms of molecular pathways activated and the cellular processes affected. We highlight the underlying complexity of these differential effects and emphasize the need to investigate various combinations of factors impacting cellular adaptation to hypoxia: total duration of hypoxia, concentration of oxygen (O_2_), and the presence of and frequency of H–R cycles. Finally, we summarize the effects of cycling hypoxia on various hallmarks of cancer highlighting their dependence on the abovementioned factors. We conclude with a call for an integrative and rigorous analysis of the effects of varying extents and durations of hypoxia on cells, including tools such as mechanism-based mathematical modelling and microfluidic setups.

## 1. Introduction

Primary tumors are rarely a cause of death in cancer patients. It is the metastatic potential of cancer cells that dictates the aggressiveness and severity of the disease and is responsible for majority of cancer-related deaths [1]. Metastasis is a complex multi-step process which involves interactions of cancerous cells with their neighboring stromal cells in the tumor microenvironment (TME). A key parameter of TME in solid tumors is hypoxia, i.e., relatively low levels of oxygen (O_2_). Hypoxia has been shown to increase the metastatic potential of cancer cells [2]. Hypoxia is not limited to TME: it also plays a crucial role during embryonic development in determining cell fate; early embryonic development happens in low oxygen environment [3]. Moreover, hypoxia has been a determining factor during evolution as organisms capable of tolerating oxidative stress and utilizing oxygen for energy production tend to have better survival fitness [4]. Thus, adaptation to hypoxia seems to be an important determinant of cellular fitness in varied physiological and pathological contexts.

Hypoxia is damaging to normal cells as well as cancerous cells. While normal cells cannot withstand prolonged hypoxia and undergo either apoptosis or necrosis [5] depending on nutrient availability along with oxygen deprivation [6], cancer cells can adapt to hypoxia by altering the expression of genes involved in various cellular processes like metabolic reprogramming, proliferation, and angiogenesis [7]—thus invoking various hallmarks of cancer [8].

In the same tumor, oxygen level typically differs in a spatio-temporal manner, creating pockets of regions with low oxygen level surrounded by regions with normal oxygen level [9,10,11]. The effect of hypoxia on cancer cells depends upon the dynamics of oxygen deprivation, based on which cellular hypoxia can be classified largely into three categories. Chronic hypoxia, also called as diffusion limited hypoxia, arises due to over-proliferation of cancer cells leading to an increased spatial cellular density, thus increasing the distance between some cells and the nearest blood vessel. Under chronic hypoxia, cells experience low oxygen tensions for prolonged periods (>24 h) which may result in cell death [7,12,13,14,15]. Acute hypoxia, also called perfusion limited hypoxia, arises due to aberrant shut down of small blood vessels often due to restriction caused by the increased cell mass or due to irregular erythrocyte flow. It is typically present for shorter timescales (few minutes to few hours) and can be reversed by regaining blood flow [7,12,13,14,15]. The third category of hypoxia—cyclic hypoxia—arises due to the transient shut down of immature, disorganized and unevenly distributed tumor vasculature resulting into periods of intermittent hypoxia which can vary from minutes to days [13,16,17,18,19,20,21]. Hypoxia followed by reoxygenation can cause “reoxygenation injury” to the cells which involves free radical formation, oxidative stress and tissue damage [7,16]. In this review, we will discuss the effect of cyclic hypoxia on the hallmarks of cancer and contrast them with those of acute and/or chronic hypoxia, with an attempt to highlight the molecular basis of these different phenomenon. Thus, the focus is on how decoding different extents of hypoxia in terms of O_2_% and in terms of duration of hypoxia can affect tumor cell behavior differently.

## 2. Tumor Hypoxia

Oxygen is an important microenvironment factor which acts as a terminal electron acceptor in the oxidative phosphorylation reaction to produce ATP. During oxidative phosphorylation, a higher risk of reactive oxygen species (ROS) production exists. Such high levels of ROS might interfere with the biochemical and physiochemical properties of cellular macromolecules, leading to cell death. Thus, maintenance of oxygen homeostasis is crucial for cell growth and survival [22]. Normal level of oxygen in healthy tissues varies widely between organs and range between ~4.6% O_2_ to 9.4% O_2_ while O_2_ concentration in tumor range on an average between 1–2% O_2_ or below [12]. Thus, tumor tissues are generally hypoxic, i.e. oxygen deprived. Tumor hypoxia was first explained by Thomlinson and Gray in 1955 where they introduced the concept of decreasing O_2_ gradient from the periphery to the center of the tumor sphere. They showed that cancerous cells grow in the periphery of the vascular stroma, while the center of larger tumor region is necrotic surrounded by intact cells which appear as rings. By evaluating the respiratory quotient of the entire tumor mass, they estimated that the center of any tumor sphere with radius greater than ~170 µm will be completely oxygen deprived (anoxic) [9]. This kind of hypoxia leads to diffusion limited or chronic hypoxia in solid tumors.

Increased oxygen demands in the growing tumor often leads to angiogenesis that forms structurally and functionally abnormal blood vessels [23] which are inefficient in blood perfusion, resulting into acute hypoxia. The consequent irregular erythrocyte flow in them may lead to cycles of hypoxia and reoxygenation which gives rise to either acute or intermittent hypoxia [19]. Brown *et al.* were the first to show the presence of acute hypoxia in mouse tumor which was produced due to intermittent opening and closing of tumor blood vessels [18]. These fluctuations in tumor perfusion were found not necessarily adjacent to the blood vessels, but also as far as 130 µm from a micro-vessel [24]. Later, the presence of cyclic hypoxia in at least 20% of tumor cells emphasized its importance as a driver of tumor aggressiveness [25].

## 3. HIF Signaling: Key Regulators of Hypoxic Response

**Figure 1:**
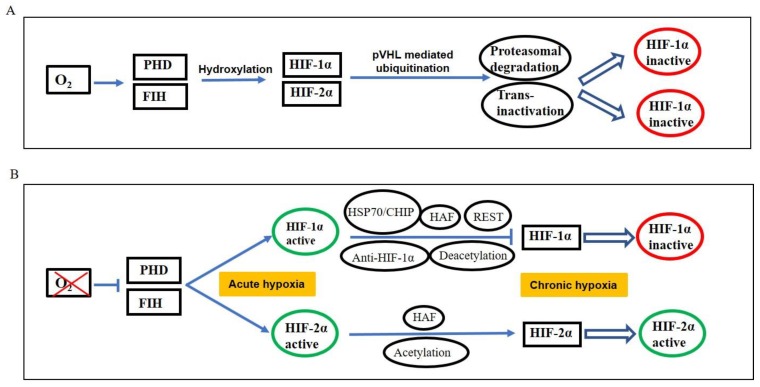
Oxygen-dependent regulation of hypoxia inducible factors HIF-1α and HIF-2α. (**A**) In presence of oxygen, HIF-1α and HIF-2α are hydroxylated by prolyl hydroxylase domains (PHDs) and FIH (Factor Inhibiting HIF-1), and then targeted for proteasomal degradation mediated by VHL protein. (**B**) Under acute hypoxia, both HIF-1α and HIF-2α are stabilized. Under chronic hypoxia, HIF-2α is stabilized while HIF-1α is downregulated.

Cells adapt to low oxygen tensions by altering expression of genes involved in cell survival and apoptosis. Hypoxia inducible factors (HIFs) are the master regulators of gene expression during hypoxia [15,22]. HIFs consist of one HIF-α subunit which belongs to basic helix–loop–helix (bHLH)/PAS (PER/ARNT/SIM) superfamily and one HIF-β subunit (also called as ARNT-aryl hydrocarbon receptor nuclear translocator). The stability of HIF-α depends on oxygen concentration, while HIF-β is constitutively expressed and is insensitive to variations in oxygen level. Under normal oxygen levels, HIF-α subunit is hydroxylated at two proline residues in the oxygen-dependent degradation domain of HIF-α by members of prolyl hydroxylase domain (PHD) family. After hydroxylation, HIF-α subunit is then recognized by von Hippel Lindau (VHL) protein [15]. VHL is a tumor suppressor protein which acts as substrate recognition molecule for an E3 ubiquitin ligase complex that targets HIF-α subunit for ubiquitination and proteasomal degradation (Figure 1A). Under hypoxia, PHD is inhibited such that HIF-α is not recognized by VHL and hence accumulates [15,26,27] (Figure 1B). The ability of HIFs to activate transcription under hypoxia is also regulated by an oxygen-regulated enzyme, FIH (Factor Inhibiting HIF-1). Under normoxic conditions, FIH hydroxylates the transactivation domain (TAD-C) of HIFs which inhibits their interaction with transcriptional co-activators p300/CREB binding proteins (CBP). Under hypoxia, FIH is inactivated, hence stabilizing HIFs [28,29]. Stable HIF-α proteins translocate into the nucleus and dimerize with HIF-β subunit. HIF-α/HIF-β heterodimers directly bind to the HIF responsive element (HRE) located in promoter of the target genes and regulate their transcription [30].

Three HIF-α proteins have been identified in higher metazoans—HIF-1α, HIF-2α (also called endothelial PAS domain protein (EPAS1)) and more recently HIF-3α. HIF-1α and HIF-2α share 48% amino acid sequence identity and have similar protein structures but have distinct target genes and are differentially expressed [31,32]. HIF-1α is ubiquitously expressed, while HIF-2α expression is more tissue specific and expressed in blood vessels, kidney, liver, pancreas, heart, lungs, intestine, and brain [15]. HIF-3α contains bHLH and PAS domains similar to HIF-1α and HIF-2α but lacks C-terminal transactivation domain [15,33]. Inhibitor PAS domain protein (IPAS) is one of the splice variants of HIF-3α which acts as dominant negative regulator of HIF-1α [34]. Interestingly, HIF3-α variants can have different and sometimes even opposite functions [35], similar to other proteins whose variants can have opposing roles [36]. Moreover, HIF-1α can regulate the levels of HIF-3α [37], thus various feedback loops formed among HIF-1α, HIF-2α, HIF-3α, and their targets may alter their dynamics during various hypoxic conditions. Under hypoxic conditions, HIFs can regulate the expression of many genes involved in metabolism, angiogenesis, erythropoiesis, proliferation, and apoptosis [22].

## 4. HIF Switch during Acute and Chronic Hypoxia

**Figure 2:**
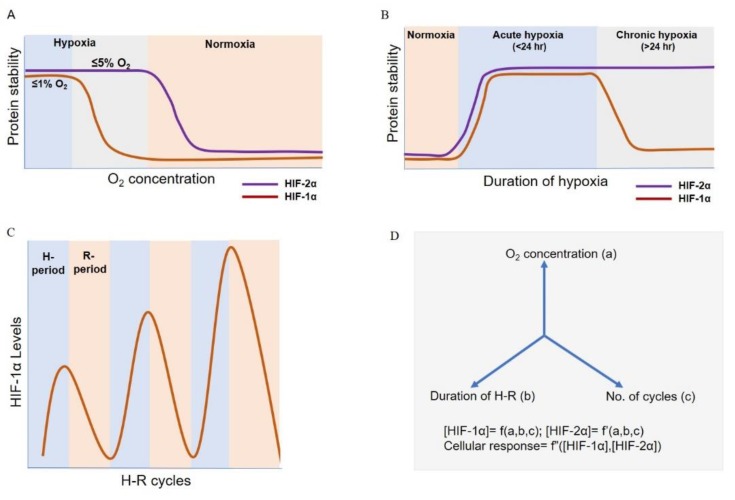
Dynamics of HIF-1α/HIF-2α stabilization under different O_2_ levels and acute, chronic and cyclic hypoxia. (**A**) Stabilization of HIF-2α over a wider range of oxygen concentration than HIF-1α. (**B**) Stabilization of HIF-1α under acute hypoxia and HIF-2α under chronic hypoxia. (**C**) Effect of H–R periods on HIF-1α levels under cyclic hypoxia. (**D**) Variables involved in HIF-1α stabilization during cyclic hypoxia.

Cells adapt to hypoxia by orchestrating coordinated sets of responses largely mediated by HIF-1α and HIF-2α, depending upon the duration of hypoxia and/or extent of hypoxia (i.e., % O_2_) (Figure 2A,B). HIF-1α and HIF-2α function in a non-redundant manner with multiple overlapping and few unique downstream gene targets [26]. In terms of oxygen concentration, HIF-2α is seen to be more stable compared to HIF-1α at higher oxygen levels (5% O_2_) in neuroblastoma cell lines SK-N-BE(2)C and KCN-69n. In these cell lines at 1% O_2_, both HIF-1α and HIF-2 α get stabilized; while HIF-1α levels stay high to mediate acute response and decay during prolonged hypoxia, HIF-2α accumulates to regulate cellular response under prolonged hypoxia [38,39]. One potential mechanism underlying this stability can be lower efficiency of hydroxylation of HIF-2α by PHD [38] and FIH-1 [29] as compared to that of HIF-1α, during higher oxygen tensions.

Besides being a function of % O_2_, HIF-1α and HIF-2α also get differentially activated depending on the duration of hypoxia. HIF-1α protein levels typically peak around 4–8 h and continuously decrease thereafter and are undetectable around 18–24 h; while HIF-2α levels are stabilized relatively later and tend to play a key role during chronic hypoxia (24–72 h) [31,38,40,41,42,43]. It should be noted that the categorization of acute and chronic hypoxia in terms of time has not been consistent across multiple in vitro or in vivo studies performed, for instance, 5.5 h has also been referred to as ‘chronic hypoxia’ [44] and 48 h treatment has been referred to as ‘acute hypoxia’ [45].

Multiple factors can be responsible for this temporal regulation of HIF switch from acute to chronic hypoxia. First, HSP-70/CHIP (a ubiquitin ligase) complex can mediate the ubiquitination and consequent proteasomal degradation of HIF-1α, but not that of HIF-2α, by directly interacting with HIF-1α under prolonged hypoxia [46,47]. Second, hypoxia-associated factor (HAF, a E3-ubiquitin ligase) has been shown to mediate ubiquitination and proteasomal degradation of HIF-1α, but not that of HIF-2α, in normal as well as hypoxic conditions [48]. Interestingly, HAF is upregulated during prolonged hypoxia and it can transactivate HIF-2α by directly binding to its C-terminal, switching the hypoxia dependent response from a HIF-1α to HIF-2α driven one [40]. Third, in human lung epithelial cells A549, both HIF-1α and HIF-2α were found to be strongly induced during acute hypoxia, but during prolonged hypoxia, HIF-1α level decreased while HIF-2α was stably maintained. The gradual decrease in HIF-1α level was attributed to a negative feedback loop in which destabilization of HIF-1α mRNA was mediated by HIF-1α specific anti-sense RNA whose expression was transcriptionally activated by both HIF-1α and HIF-2α proteins [41]. Fourth, differential chromatin modification of HIF-1α and HIF-2α under hypoxic conditions can lead to reduced mRNA levels of HIF-1α in SK-N-BE(2)C, SK-N-ER and SH-SY5Y cells. Under chronic hypoxia (1% O_2_, 24 h), acetylation of core histones (H3 and H4) in the promoter/enhancer region was decreased in HIF-1α promoter region while it was increased in HIF-2α promoter region [43]. Fifth, REST (Repressor Element 1-Silencing Transcription factor) was seen to transcriptionally repress HIF-1α specifically under prolonged hypoxia (1% O_2_, 0–48 h) by directly binding to its promoter in HEK293 cells. Knockdown of REST increased expression of HIF-1α, but not that of HIF-2α, suggesting REST to be an important factor for resolving HIF-1α dependent transcription during prolonged hypoxia [49]. More recently, crucial roles of miRNA in regulating switch from HIF-1α mediated response during acute hypoxia to HIF-2α mediated response during prolonged hypoxia has been established [50]. Importantly, miR-429 has been shown to mediate transition from HIF-1α to HIF-3α during prolonged hypoxia in endothelial cells [51]. Put together, these studies indicate various mechanisms that can mediate a switch from HIF-1α driven response during acute hypoxia to a HIF-2α/HIF-3α driven response during chronic hypoxia in a context-dependent manner (Table 1).

## 5. HIF Dynamics during Intermittent Hypoxia

Cellular response to chronic hypoxia is different from that to cyclic hypoxia (also called as intermittent hypoxia (IH)). Studies have found higher expression (stability and activity) of HIF-1α in cancer cells as well as endothelial cells during cyclic hypoxia as compared to chronic hypoxia [44,55,56,57,58,59,60] (Figure 2C). Different studies have used different durations of hypoxia–reoxygenation (H–R) cycles and percentage of O_2_ for creating cyclic hypoxic conditions. Some studies have also referred the hypoxic conditions used as ‘acute cyclic’ [61] and ‘chronic cyclic’ [62] depending on the duration of H–R cycles. Thus, the outcomes of such studies show some context-dependent variation, but some common themes emerge, such as increased HIF-1α levels (Table 2).

Toffoli *et al.* showed enhanced stability of HIF-1α protein by post-translational modification. During hypoxic pulses of the intermittent hypoxia (1% O_2_, 1 h hypoxia; 30 min reoxygenation; 4 cycles) treatment, levels of phosphorylated HIF-1α progressively increased via the activation of protein kinase A (PKA) in EAhy926 and HAMEC-1 endothelial cells [63]. But, during reoxygenation periods, neither HIF-1α protein levels nor its phosphorylated form was observed [55,63]. Interestingly, the levels of HIF-1α was found to be higher after every new cycle of hypoxia [55]. However, a comparison of HIF-1α activity (i.e., levels of HIF1-α regulated genes) during the hypoxic and reoxygenation pulses within a H–R cycle remains to be done.

During reoxygenation, while nuclear HIF-1α levels decreased, but some HIF-1α targets can be upregulated by a stress granule dependent pathway via a transcription-independent translation-dependent mechanism. Reoxygenation leads to the depolymerization of stress granules that had sequestered HIF-1α dependent gene transcripts during the hypoxia pulse [64]. This accumulation of HIF-1α regulated transcripts after reoxygenation is hypothesized to recover cells from hypoxia shock and prepare them for future insults [64]. Whether this mechanism operates in multiple H–R cycles remains to be identified. The dynamic changes in proliferative index [65] and translation-dependent mechanisms [64] during H–R cycles has been documented in multiple cell types, however, a comprehensive mapping of signaling pathways implicated in cellular adaptation to acute vs. chronic vs. cyclic hypoxia, and of the involvement of HIF-1α and/or HIF-2α, remains to be done.

Another mechanism by which HIF-1α levels can increase during cyclic hypoxia is via higher ROS levels [66]. Exposure of cells to hypoxic environment leads to depletion of cellular ATP which inhibits the energy consuming processes like translation. Reoxygenation of hypoxic cells gradually replenishes ATP levels but sudden increase in molecular oxygen results into production of ROS which causes oxidative stress in the cells. It is now well accepted that periods of reoxygenation results into higher ROS production than the hypoxic period due to availability of greater molecular oxygen [67]. The main sources of ROS are typically mitochondrial respiration and NADPH oxidase (NOX) [68]. ROS causes DNA damage, genetic instability and impairs functions of macromolecules [68]. ROS-dependent stabilization of HIF-1α has been shown to be required for HIF-1α activation under hypoxic condition [69]. *In vitro* and *in vivo* experiments done using U87 glioblastoma multiforme cells and tumors showed that experimentally imposed cyclic hypoxia (0.5–1% O_2_, 1 h hypoxia; 30 min reoxygenation; 3 cycles) increased HIF-1α signaling and enhanced its stabilization in a ROS-dependent manner [58]. Consistently, increased ROS during cyclic hypoxia (0.5–1% O_2_, 1 h hypoxia; 30 min reoxygenation; 3 cycles) upregulated HIF-1α and NF-κB expression in glioblastoma cells [60]. The mechanistic basis for ROS-mediated HIF-1α was provided by Malec *et al.* in lung adenocarcinoma cells; they showed that intermittent hypoxia (1% O_2_, 2 h hypoxia; 2 h reoxygenation; 3 cycles) increases the levels of NOX1-induced ROS. ROS upregulates nuclear factor erythroid 2-related factor 2 (NRF2; a key regulator of oxidative stress) and an antioxidative enzyme thioredoxin (TRX1) which can lead to accumulation of HIF-1α. [56]. However, in contrast to HIF-1α accumulation during intermittent hypoxia (1.5% O_2_, 30 sec hypoxia; 5 min reoxygenation, 60 cycles), HIF-2α can get downregulated via calpain-dependent degradation, resulting in increased oxidative stress [70]. Thus, intermittent hypoxia results into selective upregulation of HIF-1α instead of HIF-2α.

## 6. Chronic vs. Cyclic Hypoxia and Hallmarks of Cancer

**Figure 3:**
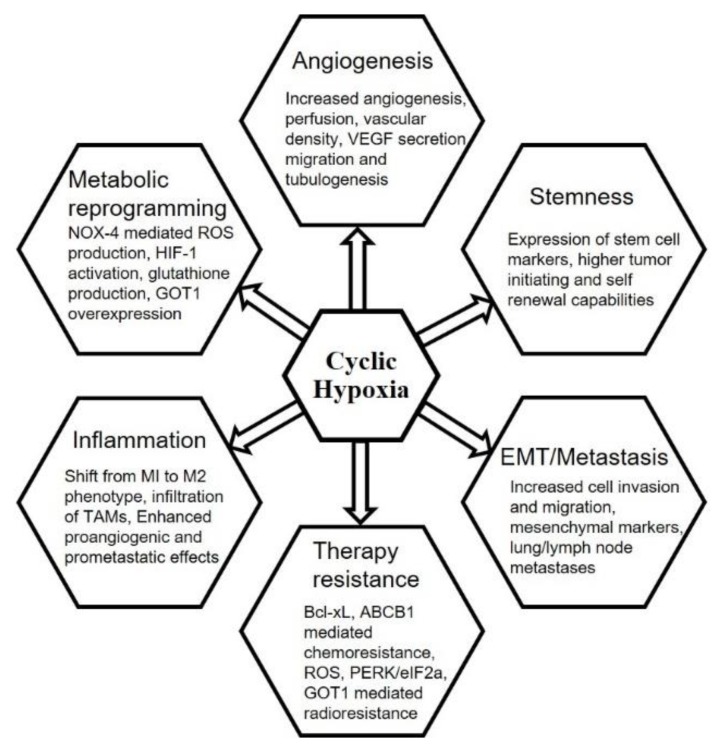
Effect of cycling hypoxia on various hallmarks of cancer.

Cellular hypoxia affects all crucial processes involved in tumor growth, invasion and metastasis. Metastasis is a multi-step complex process involving steps such as dissemination from primary tumor, entry into circulation, survival during circulation in matrix-deprived conditions and colonization of distant organs [1]. During the metastatic journey, cells may face varying extents of therapeutic attack, lack of nutrient and/or oxygen availability and other stressed conditions to which they dynamically adapt. In the next section, we will highlight effect of intermittent hypoxia on these processes for tumor progression (Figure 3).

### 6.1. Tumor Hypoxia and Angiogenesis

Under hypoxic conditions, many important factors involved in angiogenesis are stimulated via HIF-1 and HIF-2 proteins [77,78]. For instance, HIF-1 can directly enhance the transcription of erythropoietin—a key hormone that stimulates angiogenesis—under acute hypoxia (1% O_2_, 2–4 h) in Hep3B cells [78]. Angiogenesis is essential for the growth and progression of tumors [8]. Endothelial cells present in the tumor microenvironment form new blood vessels and support tumor growth by supplying oxygen and nutrients while tumor cells can support survival of endothelial cells by secreting important factors like VEGF [79].

Intermittent hypoxia (IH) affects tumor cells as well as endothelial cells and shows proangiogenic effects [80]. IH (0.5–1% O_2_, 1 h hypoxia; 30 min reoxygenation, 3–4 cycles) can increase survival of endothelial cells (EAhy926, HUVEC) under proapoptotic stimuli. Moreover, these cells showed enhanced ability to migrate and exhibit tubulogenesis in a HIF-1α dependent manner, but cells not exposed to H–R cycles (1% O_2_, 5.5 h) failed to display such phenotypes [44,57]. These studies show that HIF-1α driven intermittent hypoxia response enhances tumor angiogenesis. Further, studies using dorsal window chamber technique in A-07 human melanoma xenografts have shown that ‘acute cyclic’ hypoxia (8% O_2_, 10 min hypoxia; 10 min reoxygenation, 12 cycles) increased angiogenesis and perfusion of tumors in a VEGF-A dependent manner with increased vascular density. Although, the primary tumor growth was not affected significantly, but pulmonary metastasis increased [81,82]. In contrast, another recent study implementing H–R cycles, but at a different timescale as compared to the above-mentioned ones, arrived at a different conclusion. Liver cancer cells HepG2 and Huh7 exposed to intermittent hypoxia (1% O_2_, 24 h hypoxia, 24 h reoxygenation, 3 cycles) induced lower VEGF-A secretion as compared to a continuous hypoxia (1% O_2_, 48 h). Similarly, in vivo experiments showed lesser pro-angiogenic effects of IH treated cancer cells as compared to continuous hypoxia treated [45]. Thus, the pro-angiogenic effect of IH is likely to depend on the timescale of H–R cycles and/or the cell type (Table 3).

While IH induced angiogenic factors are now being considered to improve tissue repair and recovery [83], we still lack a rigorous and quantitative understanding of how the complex interplay between the timescale of H–R cycles, O_2_ concentration, and cell type governs the angiogenic response of cells to IH.

### 6.2. Stemness

Tumors are heterogeneous and are comprised of different cell types present in tumor microenvironments such as cancer cells, immune cells, endothelial cells, and other stromal cells. Genetic and non-genetic heterogeneity has been seen among cancerous cell population itself [84,85]. A sub-population of cancer cells has been shown to display abilities of dedifferentiation such that they can self-renew as well as give rise to multiple differentiated cells [86,87]. These cells are called cancer stem cells (CSCs) as they display stem cell-like properties demonstrated by formation of spheroids in 3D cultures. CSCs are thought of as the main cause of relapse [88], and recent studies have argued that CSCs and non-CSCs can interconvert and they are in a dynamic equilibrium [89], suggesting that ‘stemness’ is a cellular trait that can be acquired reversibly [90].

Tumor hypoxia can increase the CSC population through HIF-1α and HIF-2α protein driven responses. Specifically, under chronic hypoxia (2% O_2_, >24 h), enhanced expression of HIF-2α has been shown to promote CSC phenotype and increase tumorigenic capabilities in glioblastoma cells [91]. IH has also been reported to select for cancer cells with stem-like properties which increases survival and metastatic potential of cancer cells (Table 4). Imposing cyclic hypoxia–reoxygenation (1% O_2_, 7 days hypoxia, 1–3 weeks reoxygenation, 3 cycles), Louie *et al.* were able to expand cancer stem-like cells from breast cancer cell lines MDA-MB 231 and BCM2 and showed that as compared to parent cells, these stem-like cells were highly tumorigenic and readily formed colonies [92]. Similar observations were demonstrated in human neuroblastoma cell line NB1691 where, as compared to normoxia, intermittent hypoxia (1% O_2_, 24 h hypoxia, 24 h reoxygenation, 1, 5, or 10 cycles) suppressed cell differentiation and enhanced neural crest-like and stem-like properties in a HIF-1α dependent manner [71]. More recently, Alhawarat *et al.* expanded the CSCs of MCF-7 breast cancer cell line with elevated chemoresistance and stem-like properties by exposing them to intermittent hypoxia (1% O_2_, 8 h hypoxia, 3 times a week, 8 weeks). Moreover, the conditioned media from these CSCs enhanced angiogenesis and wound healing capabilities of HUVEC cells as compared to normoxic controls, suggesting some IH-driven paracrine communication within the tumor microenvironment [93].

A comparison of the effect of chronic vs. intermittent hypoxia on CSCs remains underexplored. Miao *et al*. showed that compared to chronic hypoxia (1% O_2_, 48 h), intermittent hypoxia (1% O_2_, 12 h hypoxia, 12 h reoxygenation, multiple cycles) resulted into a greater selection of stem/progenitor cancer cells which had enhanced self-renewal and invasive abilities in the gastric cancer cell line SGC-7901 [72]. This study suggests that intermittent hypoxia, but not necessarily chronic hypoxia, creates a microenvironment which is favorable for selection of CSCs which further contribute for the survival and metastatic growth of tumor cells. It should be noted that the functional involvement of HIF-1α and/or HIF-2α in enriching for CSCs under IH/CH remains to be conclusively demonstrated.

### 6.3. Epithelial–Mesenchymal Transition, Invasion, Migration and Metastasis

Epithelial–mesenchymal transition (EMT) is a cell biological process that can enhance the metastatic ability of cancer cells by inhibiting epithelial traits such as cell–cell adhesion and apico-basal polarity and promoting mesenchymal traits of migration and invasion. Cancer cells typically undergo EMT to disseminate from the primary tumor, invade the neighboring tissues and migrate to distant locations where they undergo mesenchymal–epithelial transition (MET)—the reverse of EMT —to form secondary tumors [98]. EMT is usually measured via levels of a cell–cell adhesion molecule E-cadherin and an intermediate filament Vimentin; it involves partial/complete loss of E-cadherin and increase in Vimentin [99]. Upregulation of HIF-1α under hypoxic conditions (1% O_2_, 18 h) has been shown to increase EMT and metastasis by directly regulating expression of TWIST, a key transcription factor involved in EMT. Co-expression of HIF-1, TWIST, and SNAIL (another EMT-inducing transcription factor) in primary tumors of head and neck cancer patients has also been correlated to higher metastasis and poor prognosis [100].

Intermittent hypoxia can also influence EMT, invasion and metastasis of cancer cells (Table 4). Pancreatic cancer cells Panc-1 and BxPC-3, when exposed to intermittent hypoxia (1% O_2_, 12 h hypoxia, 12 h reoxygenation, 5 cycles), show an EMT phenotype in a HIF-1α dependent manner [73]. Similarly, as compared to normoxia, accumulation of HIF-1α under intermittent hypoxia (1% O_2_, 12 h hypoxia, 12 h reoxygenation, 10 cycles) resulted into upregulation of vimentin and decreased cell proliferation in triple-negative breast cancer cells, MDA-MB-231. Moreover, compared to normoxia and chronic hypoxia (1% O_2_, 48 h), intermittent hypoxia showed increased migration of these cells; the greatest effect on migration—out of 5, 10, 15, and 20 IH cycles—was produced by 10 IH cycles [74]. Another in vitro analysis, conducted in medulloblastoma cells DAOY and D283, demonstrated increased invasion and migration under conditions of IH (1% O_2_, 48 h hypoxia, 48 h reoxygenation, 18–20 cycles) as compared to normoxia. Various mesenchymal markers such as SNAIL, vimentin, and *N*-cadherin were upregulated and many epithelial markers such as ZO-1, *E*-cadherin decreased [94]. However, a recent study showed opposite effects of intermittent hypoxia (1% O_2_, 8 h hypoxia, 2 to 8 h reoxygenation) on two different nasopharyngeal cancer cell lines, CNE1 and CNE2. While CNE1 cells experienced poor migration and invasion but enhanced proliferation in response to IH, CNE2 cells showed better migration but poor proliferation [95]. These differences highlight how cells can respond differently to IH, and in general, to other conditions in a tumor microenvironment. Moreover, EMT is a multi-dimensional and non-linear process with changes in apico-basal polarity, cell migration, basement membrane remodeling, cell–cell adhesion etc. [101]. Thus, identifying the molecular and morphological traits of EMT is not always unambiguous. A better characterization of the dynamics of EMT measured through single-cell experiments [102,103,104,105] may help resolve some of these potentially confounding effects of IH on EMT. In addition to EMT-associated migration modes, cells exposed to hypoxia may display other modes of migration such as amoeboid migration [106]; identifying underlying mechanisms of plasticity in migration modes [107] may be helpful in deciphering the effects of hypoxia on metastatic potential.

Multiple studies have shown that cyclic hypoxia can increase cancer metastasis more emphatically than chronic hypoxia can. For instance, intermittent hypoxia (2–7% breathing O_2_, 10 min hypoxia, 10 min reoxygenation, 12 cycles, 7 days per week) resulted into greater spontaneous lung metastases than hypoxia without any H–R cycles (2–7% breathing O_2_, 120 min continuous hypoxia, 7 days per week) in KHT murine fibrosarcoma [61]. The authors also reported greater lymph node metastasis and reduced tumor growth than normoxic control in orthotopic mouse model implanted with human cervical cancer cells, ME-180, under intermittent hypoxia (2–7% breathing O_2_, 10 min hypoxia, 10 min reoxygenation, 12 cycles, 21 days) [96]. An investigation of correlation between spontaneous metastases promoted by endogenous chronic and cyclic hypoxia in D-12 and R-18 human melanoma xenograft mouse models, revealed that although both resulted into spontaneous metastases, cyclic hypoxia showed greater extent of metastases than chronic hypoxia [108]. More recently, intermittent hypoxia (1% O_2_, 24 h hypoxia, 24 h reoxygenation, 9 days) but not chronic hypoxia (1% O_2_, 9 days) was shown to increase pro-tumorigenic cytokine secretion and higher pro-metastatic gene expression with enhanced lung metastases in human breast cancer xenograft model [97]. Put together, these observations underscore the effect of intermittent hypoxia in increasing metastasis of cancer cells as compared to chronic hypoxia at least at a phenomenological level. However, the involvement of HIF-1α and/or HIF-2α in these phenotypes, as well as any other molecular mechanisms at play, remains to be established.

### 6.4. Anti-Cancer Therapies

A small population of cancer cells in tumors tend to exhibit enhanced resistance against multiple therapies. Oxygen is a potent radiosensitizer which increases DNA damage upon irradiation mediated by free radical formation. It was observed that oxygen-deprived cells were three times more resistant to radiation therapy than well-oxygenated cells, when irradiated. As tumor microenvironment can have regions of chronic and cyclic hypoxia, they can produce persistent and/or transient radio-resistance, respectively [109]. Hypoxia can also play an important role in promoting drug-resistance by facilitating drug efflux and inhibiting pro-apoptotic signals. Many drugs require molecular oxygen for their action, hence they show a weaker effect under hypoxic conditions. Finally, drug distribution can be limited due to poor diffusion in hypoxic regions [110,111].

Cells respond to DNA damage by arresting mitotic cell cycle and repairing the damage. In case the damage is unrepairable, cells are subjected to apoptosis [112]. This process is often mediated by a tumor suppressor protein p53 which has been shown to be involved in cell cycle arrest and induction of apoptosis upon DNA damage [113]. TP53 gene mutations are very common in solid tumors and can cause genetic heterogeneity among cancer cells. Under hypoxic conditions, cancer cells carrying wild type p53 protein undergo apoptosis while small population of cells carrying mutated p53 are clonally selected which shows enhanced tolerance to hypoxia, radiation therapy and chemotherapy [114,115]. Consistently, exposure of epithelial cells to intermittent hypoxia (0.2–1% O_2_, 16 h hypoxia, 8 h reoxygenation, 50 cycles) resulted into selection of cells with reduced p53 and *E*-cadherin expression which showed increased survival, invasion and therapy resistance than normoxic control cells [116]. These molecular mechanisms suggest cells having undergone an EMT-like response; which has been associated with multidrug resistance across carcinomas [117].

(a) Resistance to Chemotherapeutic Drugs

As compared to continuous hypoxia (0.5–1% O_2_, 3 h), cycling hypoxia (0.5–1% O_2_, 1 h hypoxia, 30 min reoxygenation, 3 cycles) can induce chemoresistance against telozolomide in glioblastoma multiforme cells, U251, U87, and GBM8401 by inducing ROS formation which resulted into HIF-1α and NF-κB activation [60]. HIF-1α can upregulate efflux transporter, ABCB1, leading to greater chemoresistance against doxorubicin and BCNU in these cells. Moreover, regions of the tumor having endogenous cycling hypoxia were found to have greater chemoresistance and ABCB1 expression in xenograft models [75] (Table 5).

(b) Radioresistance

Intermittent hypoxia (7% O_2_, 1 h hypoxia, 30 min reoxygenation, 3 cycles), but not hypoxia without H–R cycles (7% O_2_, 3 h hypoxia) has been reported to exert HIF-1α dependent radioresistance in TLT-liver carcinoma xenograft models by inhibiting apoptosis in vascular as well as tumor cell compartments thus, resulted into increased tumor regrowth after irradiation. In vitro irradiation experiments using melanoma and fibrosarcoma cell lines—FsaII and B16-F10 respectively—exposed to intermittent hypoxia (<1% O_2_, 1 h hypoxia, 30 min reoxygenation, 3 cycles) showed radioresistance, but cells exposed to normoxia did not [57].

In some cases, HIF-1α levels and/or signaling have been implicated in conferring this radioresistant phenotype to cells exposed to IH. For instance, Liu *et al.* showed a HIF-1α dependent increased radiation resistance in human lung cancer cell lines, A549 and NCI-H446 upon irradiation when exposed to intermittent hypoxia (0.1% O_2_, 24 h hypoxia, 72 h reoxygenation, 20 cycles) [62]. Consistently, glioblastoma cells U87 exposed to intermittent hypoxia (0.5–1% O_2_, 1 h hypoxia, 30 min reoxygenation, 3 cycles) exerted greater radio resistance than those exposed to chronic hypoxia (0.5%–1% O_2_, 4 h hypoxia). This response was mediated by ROS induced HIF-1α protein levels. Similarly, compared to chronic hypoxia (7% O_2_, 4 h), intermittent hypoxia (7% O_2_, 1 h hypoxia, 30 min reoxygenation, 3 cycles) induced greater radiation resistance in U87 glioma xenograft models [58]. In a follow-up study, the authors showed that NOX4 (NADPH oxidase subunit 4) was a critical mediator of radio resistance exerted by cyclic hypoxia (0.5–1% O_2_, 10 min hypoxia, 10 min reoxygenation, 12 cycles) in glioblastoma cell lines, GBM8401 and U251 and tumor models. They also found higher levels of ROS, NOX-4 and radioresistance in cells facing cyclic hypoxia as compared to other subpopulations of tumor [118]. Similar observations have been made for endothelial cells as well, where translation of HIF-1 regulated mRNAs confer radioresistance to cells [64].

HIF-1α independent mechanisms may also affect the radioresistance of cells exposed to IH. For instance, Rouschop *et al.* reported that radioresistance under cycling hypoxia with severe oxygen deficiencies (<0.02% O_2_, 1 h hypoxia, 1 h reoxygenation, 2–5 cycles) in U373-MG and HCT116 cells was mediated by PERK/eIF2a arm of unfolded protein response while HIF-1α appeared non-essential. PERK/eIF2a signaling was found to exert protection against ROS by facilitating cysteine uptake and glutathione synthesis [119]. More recently, protection against ROS has also been reported to be exerted by mitochondrial citrate carrier (SLC25A1), which was shown to be upregulated under cyclic hypoxia (<1% O_2_, 48 h hypoxia, 120 h reoxygenation, 16 and 25 cycles) in NCI-H460, DU145 and T98G cell lines. Pharmacological inhibition of SLC25A1 led to increased radio-sensitivity in these cell lines [120]. In a follow-up study, the inhibition of another mitochondrial citrate carrier SLC25A10 was shown to abolish the radioresistance provided by cyclic hypoxia by increasing cytotoxic effects of irradiation. Also, clinical data analysis found a correlation between overexpression of SLC25A10 and poor prognosis in lung cancer patients [121]. Another mechanism by which cancer cells can adapt to high levels of ROS during cyclic hypoxia is via glutamic-oxaloacetic transaminase (GOT1). NCH-H460, DU145, and T98G cell lines, when exposed to cycling hypoxia (<0.1% O_2_, 48 h hypoxia, 120 h reoxygenation, 16 or 25 cycles), induced upregulation of GOT1 which rewires cellular metabolism for NADPH synthesis and glutathione regeneration using glutamine. Indicating a functional role, glutamine deprivation or GOT1 inhibition resulted in reduced glutathione levels, increased ROS levels, and restored cell death upon irradiation [122]. Thus, reduced levels of antioxidants such as glutathione [68] can render cancer cells to harmful effects of accumulated ROS. Put together, cells exposed to cyclic hypoxia that tend to enhance ROS levels typically employ mechanisms to prevent themselves from the resultant oxidative stress (Table 5). A failure to implement these safety mechanisms may lead to radio-sensitization of these cells.

### 6.5. Inflammation

Inflammation is a hallmark of cancer and an important feature of tumor microenvironment (TME) that facilitates escape of cancer cells from the immune system [8]. Hypoxic TME induces cancer cells to release a number of immunosuppressive factors which provide immune resistance and promote angiogenesis [123]. For example, severe hypoxia induces tumor cells to release IL10 and TGFβ which promote differentiation of tumor associated macrophages (TAM) to M2 macrophages that shows immunosuppressive functions [124]. Increased infiltration of CD206 + M2 macrophages in intermittent isobaric mouse model bearing TC-1 tumors supports this observation [125]. Also, TGFβ inhibits expansion and cytotoxic functions of T-cells and natural killer cells and suppresses antigen presentation function of dendritic cells [126]. VEGF and other angiogenic factors induced by hypoxia have also been shown to promote immune suppressive phenotype in tumor cells [123].

However, studies comparing the effects of acute vs. chronic vs. IH on inflammation are relatively few (Table 6). In an elaborate study, Tellier *et al.* reported that TGFα-induced endothelial cells grown under cyclic hypoxia (1% O_2_, 1 h hypoxia, 30 min reoxygenation, 4 cycles) showed enhanced proinflammatory response via over-activation of NF-κB which resulted in increased secretion of IL-6, IL-8, I-CAM (a cell adhesion molecule), and monocyte adhesion. They validated these findings in tumor bearing mice and found similar enhancement of pro-inflammatory cytokines mRNA and infiltration of leucocytes in tumors. Moreover, clinical data analysis showed a signature phenotype (BIRC5^low^/PTGS2^high^/I-CAM^high^/IL-6^high^/IL-8^high^) for cyclic hypoxia specific increased inflammation which correlated with poor prognosis in colon cancer patients, further strengthening their findings [127]. Gutche et al. showed that enhanced pro-metastatic and pro-inflammatory phenotype associated with triple-negative inflammatory breast cancer cell line SUM149PT was mediated by NF-κB overexpression induced higher ROS levels produced during cyclic hypoxia (0.2% O_2_, 1 day hypoxia, 3 days reoxygenation, 15 cycles) [128]. NF-κB mediated signaling has been implicated in generating various subsets of CSCs within a TME [90,129]; reminiscent of observations connecting IH to CSC enrichment [71]

## 7. Intermittent Hypoxia Dynamics in Obstructive Sleep Apnea (OSA)

Intermittent hypoxia has also been observed during obstructive sleep apnea (OSA)—a sleep disorder which causes periodic obstruction in breathing during sleep due to improper functioning of upper airways, and results into sleep fragmentation [130]. Patients with OSA experience rapid (15–60 s) cycles of hypoxia and reoxygenation which occur for 8–9 h during sleep and continues for weeks to months or even longer resulting into short intermittent high-frequency hypoxia [131]. Recently, it has been shown that patients with obstructive sleep apnea (OSA) are at a higher risk of developing cancer [132,133,134]. Importantly, a strong correlation between intermittent hypoxia and tumor progression has been witnessed in OSA in *in vitro* and *in vivo* models [135,136,137]. Murine models of OSA are commonly used to mimic effects of OSA and IH on tumor development in vivo by injecting cancer cells in these mice to induce tumor formation. To intermittently obstruct airways *in vivo*, two kinds of approaches are typically used: a) invasive methods such as inflatable balloon in trachea, face mask or tracheostomy, or b) non-invasive approach such as exposing the mice or rats to controlled oxygen tensions in an air-tight box [137].

Alemendros *et al.* were the first to show that intermittent hypoxia enhanced tumor growth in mice models of OSA. They injected B16F10 melanoma cells in mice, and mimicked OSA-like intermittent hypoxia by experimentally imposing a subset of these mice to chronic intermittent hypoxia (5% O_2_, 20 s hypoxia, 40 s reoxygenation, 60 cycles per h, 6 h per day for 14 days). Mice exposed to intermittent hypoxia were found to have higher tumor growth, as compared to those under normoxia [138]. Increased multiple myeloma progression was also observed when the myeloma resistant C57BL/6J strain of mice were injected with malignant cells 5TGM1 and exposed to intermittent hypoxia (10% O_2_, 2 min hypoxia, 2 min reoxygenation, 12cycles per hour, 10 h/day, for 4 weeks) as compared to normoxic controls [135].

Many studies in OSA models have elucidated the functional contribution of intermittent hypoxia in promoting increased tumor growth and metastasis (Table 7). Martinez *et al.* mimicked physiological OSA by exposing HCT116 colorectal cancer cells to rapid intermittent hypoxia (5 min 59 mmHg O_2_, 5 min 0mm Hg). Intermittent hypoxia treatment increased HIF-1α protein and mRNA levels. Moreover, expression of HIF-1α target genes involved in glycolysis, ECM modelling and hypoxia pathways were also found to be upregulated in hypoxia in an oxygen concentration-dependent manner, but not in a frequency- or duration-dependent manner [76]. In another study, the effect of IH depended more on the frequency of IH instead. Mice were treated with 2 different IH regimes (IH-1, IH-2) after implantation of B16F10 melanoma cells. IH-1 involved 12% FiO_2_ (fraction of inspired oxygen), 90 s hypoxia, 270 s reoxygenation, 10 cycles per hour, 8 h per day, and IH-2 involved 12% FiO_2_, 90 s hypoxia, 90 s reoxygenation, 20 cycles per hour, 8 h per day. IH-2 treatment showed greater tumor progression as compared to IH-1 and normoxic control [139]. This study suggests that different durations for which cells are exposed to hypoxia vs. reoxygenation within each H–R cycle can also impact IH-driven effects in tumor progression.

Crucial role of immune system in tumor progression and growth under IH has also been shown in OSA model systems. For example, Almendros *et al.* reported that TAMs extracted from IH (6% FiO_2_, 90 s hypoxia, 90 s reoxygenation, 20 cycles per hour, 12 h per day for 4 weeks) exposed mice tumors showed a shift from M1 to M2 phenotype in OSA model of lung cancer. These TAMs mediated increase in migration and extravasation of tumor cells [140], indicative of crosstalk between M2 macrophages and tumor cells [141]. M2 macrophage infiltration was also found to be enriched in regions surrounding the tumor, suggesting its pro-tumorigenic role [142]; this effect may be amplified under IH as macrophages may be induced to secrete higher levels of pro-angiogenic molecules such as VEGF-A [143]. Campillo *et al.* showed that increase in tumor growth and invasion in OSA mice model of lung cancer (LLC1) under IH, at least partly driven by activation of cyclooxygenase-2 (COX-2) which induced TAM shift to M2 phenotype [144]. Consistently, OSA patient derived monocytes were shown to induce an increased tumor-promoting microenvironment, an effect which could be reversed by restoration of adequate oxygen in vivo [145].

Cytotoxic T lymphocytes have also been shown to be inhibited by IH in OSA patients. For example, Cubillos-Zapata et al. showed that PD-L1 and PD-1 were overexpressed in OSA patient derived monocytes and CD8+ T cells, respectively. PD-L1/PD-1 crosstalk inhibited T lymphocyte activation and proliferation and suppressed cytotoxic activity of CD8+ T cells. Moreover, OSA patients showed increased population of MDSCs (myeloid cells capable of inhibiting innate and adaptive immune responses) with higher expression of RORC1 in their monocytes which promotes expansion of MDSC [146]. Higher expression of PD-L1 in tumor cells of IH (6% O_2_, 70 s hypoxia, 50 s reoxygenation, 8 h per day, for 5 weeks) treated OSA mice models of LLC cells has also been reported by Huang et al. [147]. Similarly, Akbarpour *et al.* showed that sleep fragmentation (SF) and IH (6% Fi O_2_, 90 s hypoxia, 90 s reoxygenation, 20 cycles per hour, 12 h per day till the tumor is palpable) increased tumor size and invasiveness, and IH reduced granzyme-B producing CD8+ cells in tumors of OSA TC1 mice model [148]. These changes in OSA models may be driven by EMT-associated changes; given that EMT has been shown to be immunosuppressive [149] including upregulation of PDL1 [150] and reduced T-cell infiltration [151]. Reduction in circulating iNKT cells (invariant natural killer T-cells) has also been reported in OSA patients providing a possible explanation of increased cancer risk in OSA patients [152]. Thus, OSA model system provides an important platform to study effect of IH on various aspects of tumor development.

## 8. Mathematical Modelling As a Tool to Understand the Hypoxia Response Dynamics

Activation of HIF-1α and/or HIF-2α is a complex process which depends on the rate of stabilization and degradation of the protein. Under normoxia, HIF-1α is hydroxylated by prolyl hydroxylases (PHD) and asparagyl hydrolylases (FIH) which mediates its proteasomal degradation and trans inactivation respectively. Under hypoxia, PHDs and FIHs are inhibited such that HIFα are stabilized and transactivated [22]. As discussed above, different combinations of factors can affect the patterns of HIF-1α and HIF-2α activation and eventually the dynamics of hypoxic adaptation: concentration of oxygen, duration of hypoxia, timescale of H–R phases during H–R cycles, number of H–R cycles, and total duration of time for which H–R cycle is continued.

Mathematical modelling provides a simple, cost effective and efficient way to study the crosstalk between various factors involved in HIF-1α signaling network. The first mathematical model of HIF pathway was given by Kohn *et al.* who proposed a switch like-behavior of HIF response under hypoxia and gave a comprehensive molecular interaction map (MIM) of the HIF-1α network. Their model of the ‘core network’ regulating hypoxic response included oxygen-dependent subunit of HIF-1 (i.e. HIF-1α), oxygen-independent sub-unit of HIF-1 (i.e. ARNT), an E3 ubiquitin ligase substrate recognition subunit VHL and prolyl hydroxylases PHD2 and PHD3, predicted a sharp increase in HIF-1α level under hypoxia gradually reaching a plateau phase [153]. Multiple models considering various aspects of HIFα network under changing oxygen levels have been proposed since [154,155].

Leedale *et al.* developed a mathematical model describing the effect of repetitive pulses of hypoxia on HIF-1α protein dynamics in a single cell. Their model demonstrated existence of non-monotonous relationship between the dynamics of HIF-1α transient behavior, its differential target expression and oxygen dynamics [156]. Using mathematical modelling, PHD2 was identified to be a crucial factor for HIF-1α pulsatile dynamics under hypoxic conditions and this pulsatile response was important to inhibit p53 regulated pro-apoptotic genes, thus prevented cell death [157]. Another attempt to dissect population dynamics of cancer cells under H–R cycles revealed how the relative proportions of cells under hypoxia vs. reoxygenation correlate with the number of regional lymph nodes that the cancer has spread to [158].

Thus, mathematical modelling can be an excellent approach to assess the effects of oxygen concentrations, durations and frequencies of H–R periods on tumor progression (Figure 2D). The presence of vast experimental *in vitro* and *in vivo* data showing diverse effects of IH treatments on tumor development and measuring oxygen tensions in solid tumors [19,159,160,161] will be useful in obtaining parameters and guiding us towards the next set of most useful experiments to decode the dynamics of hypoxia response.

Another tool that can help quantitively dissect the effects of acute vs. chronic vs. cyclic hypoxia is microfluidic devices that can successfully mimic TME in vitro with varying concentrations of oxygen [162,163,164]. By using 3D sprouting angiogenesis assay in microfluidic device, it was reported that as compared to normoxia, both chronic (3.2% to 4.6% steady state O_2_ gradient) and intermittent hypoxia (1.7% to 4.5% fluctuating O_2_ gradient) showed greater vessel growth in chronic hypoxia than in intermittent hypoxia. The range of oxygen tensions can be achieved by varying parameters such as oxygen scavenger concentration, time exposed to an oxygen scavenger, flow rate of the oxygen scavenger, membrane wall thickness, and number of active scavenger lines [164]. Such experiments, combined with mechanism-based mathematical models, can decipher the dynamics of HIF signaling under acute vs. chronic vs. cyclic hypoxia, and their consequent effects on cellular response [52,53,54,165].

## 9. Conclusions

In this review, we summarized hypoxic conditions used to experimentally impose hypoxia in vitro and in vivo with respect to the three broad classes of hypoxia: acute, chronic and intermittent, and the different molecular mechanisms and phenomenon that are activated in these scenarios. HIF-1α and HIF-2α are known as master regulators of cellular hypoxic responses whose levels are dependent on various factors: the duration of hypoxia, O_2_ concentration and presence of H–R cycles. We also highlighted the role of OSA model systems to study the effect of cyclic hypoxia on tumor progression. Studies involving OSA patients have provided direct evidence of the effect of cyclic hypoxia on increased tumor progression risk. Most in vitro and in vivo studies in tumor cells, endothelial cells, and in OSA models indicate a potentially crucial role of cyclic hypoxia in driving tumor aggressiveness by altering various hallmarks of cancer: inflammation, angiogenesis, metastasis, cell proliferation, and immune evasion.

These studies have involved differences in multiple parameters such as cell lines, duration of H–R, concentration of oxygen and number of H–R cycles to impose experimental hypoxia. Because of varying multiple parameters simultaneously, effect of any single parameter on the levels of HIF-1α, HIF-2α and tumor progression cannot be inferred accurately as of now. Moreover, when cells are kept at 21% O_2_ under normoxia or reoxygenation they are under hyperoxemia as 21% O_2_ is much higher oxygen tension than that present in normal physiological conditions. Additionally, the use of terms such as acute intermittent hypoxia and chronic intermittent hypoxia has been largely ambiguous across studies where the durations and cycles of H–R have not been kept consistent. To fully understand how intermittent hypoxia increases aggressiveness of tumor progression, it is important to identify the independent effects of oxygen concentration, duration of H–R and cycles of H–R on the dynamics of factors involved in HIF network. The use of integrative and quantitative approaches such as microfluidics and mathematical models can help move us towards elucidating the design principles of cellular hypoxic responses.

## Figures and Tables

**Table 1:** HIF-1α stability and activity under acute and chronic hypoxia in in vitro studies.

SN.	Cell Line	Conditions of Hypoxia	HIF-1α Stability	HIF-2α Stability	HIF-1α vs HIF-2α	Ref.
1	SK-NBE(2)	1% O_2_, 4 h and 72 h	Stabilized at 4 h, absent at 72 h	Stabilized at 4 h and 72 h	Greater HIF-2α expression at 4 h and 72 h hypoxia	[42]
2	SK-NBE(2), KCN-69n	1% and 5% O_2_, 2–72 h	Stabilized at 1 % O_2_ after 2 h then gradually decreased, undetected at 5% O_2_	Stabilized at 1% and 5% O_2_ after 2 h then gradually increased	HIF-1α stabilized under acute hypoxia, HIF-2α stabilized under chronic hypoxia	[38]
3	T24 and J82	1% O_2_, 0–48 h	Stabilized at 6 h, then gradually decreased	Stabilized at 6 h, then gradually increased	HIF-1α stabilized under acute hypoxia, HIF-2α stabilized under chronic hypoxia	[52]
4	SK-N-BE(2)C, IMR32 SK-N-ER, SH-SY5Y	1% O_2_, 24 h and 72 h	Stabilized at 24 h	Stabilized at 24 h and 72 h	HIF-1α stabilized under acute hypoxia, HIF-2α stabilized under chronic hypoxia	[43]
5	PC-3, DU145, LNCaP	1% O_2_, 2–24 h	Stabilized at 0.5–6 h, absent at 24 h	NA	HIF-1α active during acute hypoxia	[53]
6	MCF7	1% O_2_, 4–72 h	Stabilized at 4–8 h, decreased after 24 h	Stabilized at 24 h	HIF-1α stabilized under acute hypoxia, HIF-2α stabilized under chronic hypoxia	[54]
7	A549 cells	0.5% O_2_, 4 h and 12 h	Stabilized at 4 h, then gradually decreased	Stabilized at 4–12 h	HIF-1α stabilized under acute hypoxia, HIF-2α stabilized under chronic hypoxia	[41]
8	HEK-293, MCF7, MDA-MB-231, MCF10A	1% O_2_,0–72 h	Stabilized at 4–16 h, then gradually decreased	NA	NA	[49]

h—hour, H—hypoxia, R—reoxygenation, NA—not available.

**Table 2:** HIF-1α stability and activity under intermittent hypoxia in in vitro studies.

SN.	Cell Line	Intermittent Hypoxia (IH) (O_2_ Level, H-Duration, R-Duration, no. of Cycles)	Chronic /Continuous Hypoxia (CH) (O_2_ Level, H-Duration)	HIF-1α Stability	IH vs CH	Mechanism of HIF-1α Activation	Ref.
1	EAhy9, HUVEC	1%, 1 h, 30 min, 4	1%, 5.5 h	Stabilized during hypoxia and degraded during reoxygenation	Greater migration and tubulogenesis of endothelial cells under IH	NA	[44]
2	HUVEC	<1%, 1 h, 30 min, 3	<1%, 3 h	Progressively^@^ stabilized and accumulated during hypoxia; degraded during reoxygenation	Higher stability and activity of HIF-1α under IH	Mitochondrial respiration/ PI3K/AKT	[55]
3	A549	1%, 2 h, 2 h, multiple cycles for 6 h	1%, 6 h	Stabilized, highest HIF-1α levels after third hypoxia period of IH	NA	NOX1/NRF2	[56]
4	HUVE, BAOEC	0.5–1%, 1 h, 30 min, 3	<1%, 3 h and 6 h	Progressively^@^ stabilized and accumulated during hypoxia; degraded during reoxygenation	NA	NA	[57]
5	U87	0.5–1%, 1 h, 30 min, 3	0.5–1%, 4 h	Stabilized after 3 H-R cycles	Prolonged HIF-1α activity under IH	ROS dependent	[58]
6	PC12	1.5%, 30 s, 4 min, 60	1.5%, 1 h	Stabilized after 60 H-R cycles	Higher HIF-1α activity under IH	Transactivation by CaM kinase	[59]
7	U251, U87	0.5–1%, 1 h, 30 min, 3	0.5–1%, 3 h	Stabilized after 3 H-R cycles	Greater induction of Bcl-XL by HIF-1α under IH	ROS dependent	[60]
8	EAhy9, HMEC-1	1%,1 h, 30 min, 4	1%, 5.5 h	Progressively^@^ increase in phosphorylated HIF-1α. Highest expression at the 4th hypoxia period	PKA mediated phosphorylation of HIF-1α under IH	PKA	[63]
9	NB1691	1%, 24 h, 24 h, 10	1%, 24 h	Stabilized after 10 H-R cycles	Greater HIF-1α and HIF-2α stabilization under IH	NA	[71]
10	SGC-7901	1%, 12 h, 12 h, for 168 h	1%, for 168 h	Stabilized between 48 h and 168 h	Greater nuclear HIF-1α intensity, GLUT-1 and OCT-4 expression under IH	NA	[72]
11	Panc-1, BxPC-3	1%, 12 h, 12 h, 5	NA	Highest levels at 72 h	NA	NA	[73]
12	MDA-MB-231	1%, 12 h, 12 h, 2	1%, 48 h	Stabilized during hypoxia and degraded during reoxygenation	Greater migration under IH	NA	[74]
13	U87, GBM8401	0.5–1%, 1 h, 30 min, 3	1%, 4 h	Stabilized (assayed after 3 cycles of H-R)	Greater stability and activity of HIF-1α under IH	NA	[75]
14	HCT116	5 min 59 mmHg O_2_, 5 min 0 mm Hg, for 6 h or 18 h	4 mmHg O_2_, 6 h or 18 h	Stabilized (assayed after 6 h of H-R)	Greater stability of HIF-1α under CH	NA	[76]

h—hour, H—hypoxia, R—reoxygenation, NA—not available, @—assayed after each H–R cycle.

**Table 3:** Effect of intermittent hypoxia (IH) on angiogenesis.

SN.	Cell Line/Mouse Model	Intermittent Hypoxia	Chronic Hypoxia	Effect of IH	Ref.
1	A-07 xenograft model ^#^	8% O_2_, 10 min H; 10 min R, 12 cycles, once per day, 7 days per week till tumor volume reached 100 µm	NA	Increased angiogenesis, perfusion, vascular density	[81]
	A-07 ^$^	10–100 ppm O_2_, 30 min H, 30 min R, 6 cycles	10–10 ppm O_2_, 6 h	Increased VEGF secretion but no effect on lung metastasis	
2	EAhy926, HUVEC, BAOEC ^$^	0.5–1% O_2_, 1 h H; 30 min R, 3–4 cycles	1% O_2_, 5.5 h	Increased migration and tubulogenesis, increased survival under proapoptotic stimuli	[57], [44]

h—hour, H—hypoxia, R—reoxygenation, NA—not available, #—in vivo, $—in vitro.

**Table 4:** Effect of IH on stemness, epithelial–mesenchymal transition (EMT) and metastasis.

SN.	Cell line/Mouse Model	Intermittent Hypoxia	Chronic Hypoxia	Effect of IH	Important Markers	Ref.
1	MDA-MB-231 and BCM2 ^$^	1% O_2_, 7 days H, 1–3 weeks R, 3 cycles	NA	Expansion of stem like cancer cells (CD44^+^/CD24^-^/ESA^+^) with high tumor initiating capability, metastasis and EMT	CH44, CD24, ESA, CDH1, SNAIL, SLUG, TWIST, miR200c, miR205	[92]
2	NB1691 ^$^	1% O_2_, 24 h H, 24 h R, 1, 5 or 10 cycles	NA	Enhanced stem like properties with suppressed differentiation	VEGF, OCT4, CD133, ID-2, HES1, c-Kit, Notch1, NPY, HASH-1, dHAND, Neu N, NF-M	[71]
3	SGC-7901 ^$^	1% O_2_, 12 h H, 12 h R, 48 h	1% O_2_, 48 h	Increased stem-like/progenitor properties with enhanced self-renewal, invasion and EMT	GLUT1, CDH1, α-SMA, OCT4	[72]
4	Panc-1 and BxPC-3 ^$^	1% O_2_, 12 h H, 12 h R, 5 cycles	NA	Increased stem like cells with increased EMT, invasion, migration and autophagy	CD133, CDH1, Vimentin, CDH2, OCT4, SOX2, Beclin-1, ATG-5, LC3-II, LC3-1	[73]
5	MCF-7 and HUVEC ^$^	1% O_2_, 8 h hypoxia, 3 times a week, multiple shots	1% O_2_, 72 h, once per week	Expansion of stem like population with elevated chemoresistance and capability to induce angiogenesis	CD44, CD24, VEGF	[93]
6	MDA-MB-231 ^#^	1% O_2_, 12 h H, 12 h R, 10 cycles	1% O_2_, 48 h	Increased migration and vimentin expression	Vim.	[74]
7	DAOY, D283 and HMEC ^#^	1% O_2_, 48 h H, 48 h R, 18–20 cycles	1% O_2_, 48 h	Enhanced EMT, cell invasion, migration and angiogenesis	SNAIL, Vim., CDH2, CDH1, Zo-1	[94]
8	CNE1 and CNE2 ^#^	0.1% O_2_, 8 h H, 2 to 8 h R	NA	Increased cell proliferation and decreased invasion	NA	[95]
9	KHT murine fibrosarcoma ^#^	2–7% breathing O_2_, 10 min H, 10 min R, 12 cycles, 7 days per week	5–7% O_2_ for 2 h	Greater spontaneous lung metastases	NA	[61]
10	ME-180 xenograft mouse model ^#^	7% breathing O_2_, 10 min H, 10 min R, 12 cycles, 21 days	NA	Greater lymph node metastasis and reduced tumor growth	NA	[96]
11	PyMT-WT Luciferase/Cherry cells ^#^	1% O_2_, 24 h H, 24 h R, 9 days	1%O_2_, 9 days	Higher tumor initiating capability and metastatic potential	VEGF, MMP2, MMP9, HIF1, Aldh1, Pai, ELF5, GATA3, CH24, CH44, CD14, SCA1	[97]

h—hour, H—hypoxia, R—reoxygenation, NA—not available, #—in vivo, $—in vitro.

**Table 5:** Effect of IH on anti-cancer therapies.

SN.	Cell Line/Mouse Model	Intermittent Hypoxia	Chronic Hypoxia	Effect of IH	IH vs CH	Ref.
1	U251 and U87 ^$^	0.5–1% O_2_, 1 h H; 30 min R; 3 cycles	0.5–1% O_2_, 3 h	Resistance to temozolomide treatment mediated by Bcl-xL	Greater chemoresistance under IH	[60]
2	U87 and GBM8401 ^$^	0.5–1% O_2_, 1 h H, 30 min R, 3 cycles	1% O_2_ for 4 h	Resistance to doxorubicin and BCNU treatment mediated by ABCB1	Greater chemoresistance under IH	[75]
3	TLT xenograft model ^#^	7% O_2_, 1 h H, 30 min R, 3 cycles	7% O_2_, 3 h	Increased radioresistance and tumor regrowth	Greater tumor cell and vasculature Radioresistance under IH	[57]
	FsaII and B16-F10 ^$^	<1% O_2_, 1 h H, 30 min R, 3 cycles	NA	Increased radioresistance	NA	
4	A549 and NCI-H446 ^$^	0.1% O_2_, 24 h H, 72 h R, 20 cycles	0.1% O_2_, 16 h	Radioresistance promoted by increased S phase proliferation	Greater radioresistance shown by IH conditioned cells	[62]
5	U87 cells ^$^	0.5–1% O_2_, 1 h H, 30 min R, 3 cycles	0.5–1% O_2_ for 4 h	Increased radioresistance	Greater radioresistance shown by IH conditioned cells	[58]
	U87 xenograft with regulatable HIF-1 ^#^	7% O_2_, 1 h H, 30 min R, 3 cycles	7% O_2_, 4 h	Increased radioresistance and tumor regrowth	Greater surviving fraction and tumor regrowth in mice treated with IH	
6	GBM8401 and U251 ^$^	0.5–1% O_2_, 10 min H, 10 min R, 12 cycles	0.5–1% O_2_, 4 h	Radioresistance mediated by NOX4	Greater NOX4 induction and ROS production under IH	[118]
7	U373-MG and HCT116 ^$^	<0.02% O_2_, 1 h H, 1 h R, 2–5 cycles	NA	Radioresistance mediated by PERK/eIF2a	NA	[119]
8	NCI-H460, DU145 and T98G ^$^	<1% O_2_, 48 h H, 120 h R, 16 and 25 cycles	NA	Radioresistance mediated by SLC25A1	NA	[120]
19	NCI-H460, DU145 and T98G ^$^	<1% O_2_, 48 h H, 120 h R, 16 and 25 cycles	NA	Radioresistance mediated by SLC25A10	NA	[121]
10	NCH-H460, DU145 and T98G ^$^	<0.1% O_2_, 48 h H, 120 h R, 16 or 25 cycles	NA	Increased radioresistance mediated by GOT1	NA	[122]

h—hour, H—hypoxia, R—reoxygenation, NA—not available, #—in vivo, $—in vitro.

**Table 6:** Effect of IH on inflammation.

SN.	Cell Line/Mouse Model	Intermittent Hypoxia	Chronic Hypoxia	Effect of IH	Ref.
1	EAhy926 and HUVEC ^$^	1% O_2_, 1 h H, 30 min R, 4 cycles	1% O_2_, 6 h	Amplified proangiogenic phenotype, higher THP-1 monocyte adhesion through NF-κB	[127]
	LLC mouse model ^#^	7% O_2_, 1 h H, 30 min R, 3 cycles	NA	Enhanced inflammation in tumors with increased PTGS-2, IL-6, CXCL1 and macrophage inflammatory protein	
2	SUM149PT ^$^	0.2% O_2_, 1-day H, 3 days R, 15 cycles	NA	NF-κB mediated enhanced expression of prometastatic and proangiogenic factors	[128]
3	TC1 mouse model ^$^	Decreasing oxygen pressure (pO_2_-84 mmHg) for 4 h followed by slow recovery under normal air, 30 days	NA	Increased protumor effects of TAMs mediated by NRP-1, increase in NRP-1 levels and infiltration of CD206+ macrophages in tumor	[125]

h—hour, H—hypoxia, R—reoxygenation, NA—not available, #—in vivo, $—in vitro.

**Table 7:** Role of IH in aggressiveness of cancer from obstructive sleep apnea (OSA) model studies.

SN.	Cell Line/Mouse Model	Intermittent Hypoxia	Chronic Hypoxia	Effect of IH	Ref.
1	OSA mouse model of melanoma (B16F10) ^#^	5% O_2_, 20 s H, 40 s R, 60 cycles per h, 6 h per day for 14 days	NA	Enhanced tumor growth	[138]
2	5TGM1 ^$^	1.5% O_2_, 30 s H, 4 min R, 60 cycle	1.5% O_2_, 1 h	Reduced growth and proliferation	[135]
	OSA mouse model of myeloma resistant cells (5TGM1) ^#^	10% O_2_, 2 min H, 2 min R, 12cycles per h, 10 h/day, for 4 weeks	NA	Increased multiple myeloma progression with bone marrow engraftment	
3	HCT116 ^$^	5 min 59 mmHg O_2_, 5 min 0mm Hg, 6 h or 18 h	4 mmHg O_2_, 6 h or 18 h	Stabilization and activation of HIF-1α in dose dependent manner	[76]
4	RENCA, HAEC ^$^	1% O_2_, 30 s H, 30 s R, 24 h	NA	Increased VEGF secretion by RENCA cells	[143]
	OSA mouse model of kidney adenocarcinoma (RENCA cells) ^#^	5% O_2_, 20 s H, 40 s R, 60 cycles per h, 6 h per day for 14 days	NA	Increased angiogenesis and macrophage infiltration	
5	B16F10 and TC1 ^$^	5% O_2_, 30 min H, 30 min R, 48 h	NA	Increased tumor proliferation in presence of RAW 264.7	[140]
	OSA mouse model of lung cancer (TC1) ^#^	6% FiO_2_, 90 s H, 90 s R, 20 cycles per h, 12 h per day for 4 weeks	NA	Shift from M1 to M2 phenotype which increased invasion and migration of tumor cells	
6	OSA mouse model of lung cance (TC1) ^#^	6% FiO_2_, 90 s H, 90 s R, 20 cycles per h, 12 h per day for 4 weeks	NA	Higher macrophage infiltration, differential effect of IH on adipose tissue	[142]
7	LLC1 and RAW 264.7 ^$^	1% O_2_, 30 s H, 30 s R, 24 h	NA	Increased PGE2 secretion	[144]
	OSA mouse model of lung cancer (LLC1) ^#^	5% O_2_, 20 s H, 40 s R, 60 cycles per h, 6 h per day till tumor was palpable	NA	COX-2 dependent shift from M1 to M2 phenotype, higher growth and invasion of tumor cells	
8	Monocytes derived from OSA patients and healthy individuals ^$^	3% O_2_, 5 min H, 10 min R, 12 cycles, 4 h	NA	Tumor promoting environment mediated by VEGF in HIF-1α dependent manner	[145]
9	Monocytes and T cells derived from healthy individuals ^$^	3% O_2_, 5 min H, 10 min R, 12 cycles, 4 h	NA	PD-L1 overexpressed in monocytes, PD-1 overexpressed in CD8+ T cells	[146]
	OSA mice model ^#^	5% O_2_, 20 s H, 40 s R, 60 cycles per h, 6 h per day till tumor was palpable	NA	PD-L1 overexpressed in monocytes, PD-1 overexpressed in CD8+ T cells	
10	OSA mouse model of lung cancer (LLC) ^#^	6% O_2_, 70 s H, 50 s R, 8 h per day, for 5 weeks	NA	Increased expression of HIF-1α and PD-L1 with positive correlation	[147]
11	OSA mouse model of lung cancer (TC1) ^#^	6% FiO_2_, 90 s H, 90 s R, 20 cycles per h, 12 h per day till the tumor is palpable	NA	Increased tumor growth and invasion, reduced granzyme-B producing CD8+ cells, increased OCT4+ CSC population	[148]

h—hour, H—hypoxia, R—reoxygenation, NA—not available, #—in vivo, $—in vitro.
